# Syndemic Framework Evaluation of Severe COVID-19 Outcomes in the United States: Factors Associated With Race and Ethnicity

**DOI:** 10.3389/fpubh.2021.720264

**Published:** 2021-09-20

**Authors:** Christopher Williams, Sten H. Vermund

**Affiliations:** ^1^Department of Social and Behavioral Sciences, Yale School of Public Health, New Haven, CT, United States; ^2^Department of Epidemiology of Microbial Diseases, Yale School of Public Health, New Haven, CT, United States; ^3^Department of Pediatrics, Yale School of Medicine, New Haven, CT, United States

**Keywords:** COVID-19, syndemic risk, social determinants, systemic racism, public health infrastructure, public policy

## Abstract

Socially and economically disadvantaged racial and ethnic minorities have experienced comparatively severe clinical outcomes from the coronavirus disease (COVID-19) pandemic in the United States. Disparities in health outcomes arise from a myriad of synergistic biomedical and societal factors. Syndemic theory provides a useful framework for examining COVID-19 and other diseases that disproportionately affect vulnerable populations. Syndemic models ground research inquiries beyond individual clinical data to include non-biological community-based drivers of SARS-CoV-2 infection risk and severity of disease. Given the importance of such economic, environmental, and sociopolitical drivers in COVID-19, our aim in this *Perspective* is to examine entrenched racial and ethnic health inequalities and the magnitude of associated disease burdens, economic disenfranchisement, healthcare barriers, and hostile sociopolitical contexts—all salient syndemic factors brought into focus by the pandemic. Systemic racism persists within long-term care, health financing, and clinical care environments. We present proximal and distal public policy strategies that may mitigate the impact of this and future pandemics.

## Introduction

In the United States as of early August 2021, coronavirus disease caused by severe acute respiratory syndrome coronavirus-2 (SARS-CoV-2) has infected over 35.3 million people and is implicated in over 620,000 deaths (1). These figures, once estimated to have peaked, are again on an upward trajectory. Furthermore, an emergent body of clinical and pathologic findings provide evidence of “long-haul” COVID-19 persistent symptoms that sometimes include long-term damage of the heart, liver, kidney, as well as the blood and immune systems (2, 3). COVID-19 continues to have the most severe impact in socially and economically disadvantaged communities in the United States, even as the number of people who have been vaccinated increases. Epidemiological evidence indicates that Black, Indigenous, and People of Color (BIPOC) are more likely to get infected with SARS-CoV-2 and are at increased risk of hospitalization and death relative to Whites (4–6). During the early months of the outbreak, virologists considered whether racial differences in case numbers and case fatality rates in racial and ethnic minority communities might be attributable to genetic predisposition, biological, or pathophysiological characteristics of the hosts (7, 8). To date, no compelling mechanism has been offered to support a biological or genetic origin of disparities in racial/ethnic subgroup incidence or outcomes (9). Rather, a confluence of social determinants (5, 10), underlying comorbidities including hypertension (11), diabetes (12), obesity (13), and racial/ethnic health disparities (14–16) are key contributors to adverse outcomes in COVID-19.

Poverty ranks among the most salient drivers of health inequities and is a topic of intense investigation across diverse academic and clinical disciplines. Given suboptimal safety nets, unemployment and public facing low-wage employment are serious public health concerns (17, 18). Increased risk of COVID-19 mortality is associated with residing in high-density, multigenerational homes (17), insufficient access to primary preventative healthcare (19), speaking English as a second language, and undocumented immigration status (20, 21). A lack of preparedness has left many Americans vulnerable, however BIPOC have had to protect themselves against the virus while simultaneously facing historical and current inequalities along with hostile sociopolitical systems most apparent in racialized police brutality (22, 23).

The pandemic has brought renewed attention to service gaps in our social safety net services, intractable racial health disparities (14–16), and an erosion of public health infrastructures in the US (24). To address the multiplicity of factors that drive COVID-19 health disparities, a consensus has emerged that clinical and public health pandemic responses should not be conducted in technocratic silos, but instead within the context of a broad set of social determinants. Syndemic theory is an ecological construct that bridges disparate themes, often involving concurrent epidemics, that interact to increase disease burdens substantially. A comprehensive framework can examine both clinical and societal drivers of adverse disease outcomes (25–27). The application of syndemic theory comes with an increasing recognition that overlapping epidemics are not merely additive, but are instead synergistic and multiplicative (27). One integrative syndemic model considers COVID-19 and the psychosocial and structural burdens faced by people living with HIV, suggesting that biomedical, behavioral science, and healthcare disciplines fail to engage, reducing effectiveness of prevention and care interventions (26).

**Figure 1:**
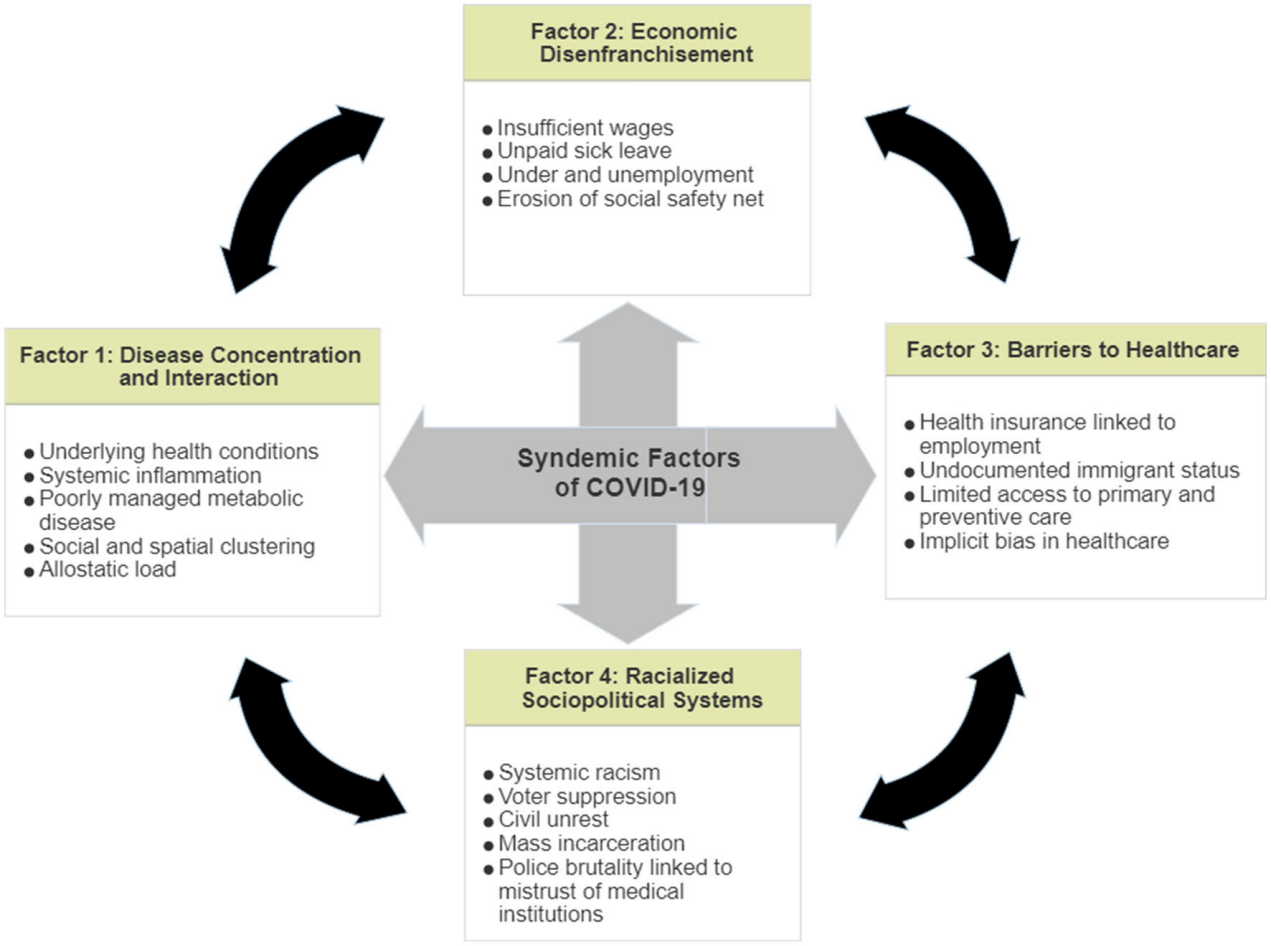
COVID-19 syndemic factors.

Syndemic theory provides an empirically-supported framework to examine the interaction between COVID-19 infections and underlying social inequities and prompts researchers to examine structural factors—housing, transportation, economic needs, mistrust of authorities and institutions—that compound the disease burden in BIPOC communities. Given that case fatality rates in the US are high when compared to other high-income countries (28), a syndemic model can be used to examine racial disparities as potentially more uniquely applicable to the US. Effective evidence-based public health practices must be integrative, and informed by syndemic approaches that recognize the synergistic effect of infectious disease, underlying chronic conditions (11–13), and sociopolitical forces including chronic systemic racism (22, 23) (Figure 1). Primary among the factors that drive COVID-19 mortality in minority communities are: disease concentration and interaction; economic disenfranchisement; primary preventative healthcare barriers; and, racialized sociopolitical systems. In this *Perspective*, we examine syndemics and its utility for structuring future analyses of both clinical and non-clinical drivers of SARS-CoV-2 infection risk and disease severity.

## Disease Concentration and Interaction

Relative to similarly high-income countries, infections and deaths attributable to COVID-19 is disproportionately high in this US (28). Acknowledging delays in systematically collecting and/or reporting COVID-19-related death by race and ethnicity in many states (29), a discernable pattern indicates that BIPOC are at higher risk for COVID-19 infection compared to Whites. The US Centers for Disease Control and Prevention (CDC) data from January 1, 2020 to July 16, 2021 indicate that CFRs for Blacks are 2.0 times higher compared to Whites (30). This grim reality is similar for Latinos/Latinas (Hispanics) whose fatality rate was 2.3 higher than that of Whites. Death rates for BIPOC have been considerably higher than that for Whites in all age categories except for persons 85 years of age and older when differences abate (31). Part of these racial/ethnic disparities are driven by underlying chronic health conditions associated with higher COVID-19-related hospitalizations and mechanical ventilation, more severe pneumonias, and lengthier time to recovery (6).

Diabetes, hypertension, renal disease, obesity, and chronic lung disease are significant predictors of more severe COVID-19 disease outcomes, especially when the chronic conditions are not well-managed (32). Syndemic theory posits that structural conditions—adverse economic, social, and healthcare circumstances—exacerbate chronic illnesses resulting in a *concentration of disease* in minority communities (27), and worsen outcomes for individuals with chronic conditions and long-term stress (26, 28). Allostatic load is a marker of chronic stress and reflects the consequences of disease concentration and interaction; it can be measured to estimate accumulation of associated physiological damage in daily life (33). This type of “weathering” is independently implicated in health disparities through the process of cumulative physiologic dysregulation for BIPOC (34).

## Economic Disenfranchisement

During the first year of the pandemic in the US, some observers asserted that the pandemic would be the “great equalizer” between those who enjoy privilege and those who do not (35). Epidemiological data reveal that the burden of the pandemic is not equal and has further perpetuated long-standing inequalities. The working poor have been driven more deeply into poverty and many low-wage jobs have accelerated toward obsolescence in response to government mandates to reduce viral transmission by closing in-person businesses (36, 37). More than 40 million people applied for unemployment financial assistance during the pandemic, a historic high not seen since the Great Depression of the 1930s. The financial toll for socioeconomically disadvantaged BlPOC has been especially devastating (36). With unemployment among Blacks being approximately twice that of Whites, the pandemic worsens deeply-embedded economic inequality (36). Structural inequities in the US labor market mean that income and other economic gains from the recovery will not be dispersed evenly across racial/ethnic groups. Labor recovery trend data indicate that workers from racial minority communities are being rehired at significantly slower rates—especially Blacks—compared to their White counterparts (37). Without sufficient employment, BIPOC are ill-equipped to buffer themselves against the socioeconomic health burden of a global pandemic.

Both underlying chronic health conditions and long-standing economic-environmental drivers compound risk for infectious diseases. These may include cardiopulmonary stressors such as exposure to adverse environmental hazards (e.g., air pollution, toxic waste), and densely-populated communities, all associated with increased risk of COVID-19 infection and consequent hospital admission (5). BIPOC are less likely to be employed in jobs that are suitable to working from home. Instead, they often travel significant distances using public transportation for low-wage work. Racial and ethnic minorities in the US are also more likely to be employed in occupations that require high contact with the public, including critical retail, trade, transportation, and social service positions (38). Though essential work drives a functioning economy, low-paid workers are less likely to receive occupational hazard protections, health benefits, paid sick leave, or compensation equal to a livable wage.

## Barriers to Primary Preventative Healthcare

A significant number of Americans do not have access to basic primary preventative healthcare, even though ≈18% of the U.S. gross domestic product is spent on healthcare (19). The implementation of the Affordable Care Act (ACA) in 2010 increased the number of low-income and poor Americans who now have access to the Medicaid-based healthcare system. However, there are still ≈27 million people who remain uninsured (39), and a disproportionate share of this group is BIPOC (4, 20), driven largely by the failure of many states to expand Medicaid. The *public charge rule* makes this situation worse for undocumented persons who fear that seeking healthcare through Medicaid or any use of public assistance, including food and housing aid, will disqualify them from receiving legal resident status. During the COVID-19 health crisis, the uninsured and the underinsured delay care and may seek help only when their disease reaches an advanced stage (40). The political wrangling and refusal of some states to expand Medicaid coverage to the working poor is an especially harmful syndemic force multiplier—made worse during a global pandemic.

The partisan divide is illustrated in the state of Missouri. In 2020, voters approved a ballot measure adding Medicaid expansion to the Missouri constitution. However in 2021, the state Governor refused to expand Medicaid to cover the working poor, a decision that was negated on July 22, 2021 by the Missouri Supreme Court ruling that the state Constitutional amendment did not violate the state constitution. The timeline for Medicaid expansion is uncertain at the time of this writing (August 2021).

Seemingly intransigent barriers to healthcare access are further demonstrated in distribution of the COVID-19 vaccine. Early reports indicate that White and high-income people were vaccinated even before persons in higher risk categories (41). This may be due to patterns of inequality that deny healthcare access, health communication strategies that do not sufficiently address vaccine hesitancy in minority communities that historically distrust healthcare providers, and logistical challenges in negotiating Internet vaccine appointment systems, and accessing vaccine centers due to transportation barriers (42). Several months after the availability of authorized vaccines, lagging distribution and uptake in BIPOC communities persists which further exacerbates COVID-19 disparities and merits intensive community engagement.

The US healthcare system is host to many of the same sociopolitical drivers of inequity that exist outside of clinical settings. One such driver is implicit bias, a type of racial prejudice that manifests in stereotypes, beliefs, or attitudes held by those in positions of authority. Often through the process of implicit bias, healthcare providers deny Black COVID-19 patients in emergency departments and intensive care units rigorous treatment and interventions offered to their White counterparts (43). This cannot entirely explain COVID-19 case fatality differences by race, but may be a contributing factor. When hospitals and providers are overwhelmed, biases may affect how scarce resources are allocated. For Black patients of higher socioeconomic status, their relative wealth confers less protection as they, too, are exposed to the negative effects of implicit bias (44).

## Racialized Sociopolitical Systems

BIPOC are chronically exposed to sociopolitical systems that perpetrate racist laws and policies, including practices that diminish access to optimal housing, education, employment earnings and benefits, consumer credit, healthcare, and equal justice (45). Exposure to a racially biased economic system is itself a toxic pre-existing condition that may predispose COVID-19 patients to severe disease (46). Racialized sociopolitical elements converge to compound racial and ethnic health inequities.

The American law enforcement system is an important archetype of structural racism. Its impact in BIPOC communities is often an unrecognized social determinant of health that ultimately threatens public health concern (47). Hostile and violent encounters with the police are linked to poor mental health among BIPOC and lead to fear and mistrust of law enforcement and other authority figures including healthcare providers and medical institutions. Mistrust of medical authority has been implicated in disproportionately high rates of COVID-19 cases and deaths among Blacks and other marginalized groups (48). Across the US, BlPOC are chronically exposed to law enforcement systems that are driven by racial animus, made evident by the death of many Blacks at the hands of police for minor offenses or suspicions. Police disproportionately use deadly force in minority communities; Black men are 2.5 times more likely than White men to be killed by police (49). Black people killed by law enforcement officers are twice as likely as Whites to have been unarmed (48). Latinos are also at increased risk of being mortally wounded by the police (50). The baseline condition for BIPOC is a perpetual state of fear, grief, and exhaustion with syndemic conditions and threats that, on balance, apply far less to Whites.

Menacing police surveillance is a toxic stress shown to predict depression, anxiety, and mental health problems (23, 47–50). Gravlee writes that “some overlapping epidemics have synergistic effects due to […] social, economic, and power inequities that shape the distribution of health (27).” Diminished personal agency to protect themselves against police brutality can seep into other elements reduced agency, as in seeking of healthcare or vaccination. Increasingly, throughout the US, remedies available through the ballot box to remediate social and racial injustice are being dismantled through the widespread passage of voter suppression laws that disproportionately affect BIPOC.

## Using a Syndemic Framework to Prepare for the Next Pandemic: A Call to Action

Public health advisories to practice social distancing are poorly suited for marginalized community residents who live in high-density residential quarters and often are employed in high-contact, low-wage work. For BIPOC who are essential workers, shelter-in-place orders are not practical and do not protect them as with most white-collar employees, and mitigation of risk must consider real-world challenges (e.g., low digital access and literacy). Public health messaging may be met with skepticism in BIPOC communities as choosing between work to support basic needs or staying at home to reduce exposure to the virus is no choice at all. Along with rebuilding of the US public health infrastructure and workforce, funds allocated for essential workers to receive hazard pay, free personal protective equipment, and safer transportation options are needed. Evidence-based public health strategies are indeed effective but must be tailored for people who do not possess privilege of place.

Equitable and data-driven allocation of scarce resources during a pandemic should be priority one. We suggest proximal and distal solutions (Table 1). A first step is to reduce the concentration of disease and underlying inequities in marginalized communities. A path forward involves public/private partnerships to improve broadband access for greater uptake of telemedicine, education, and vaccine access. Reducing air pollutants and toxic waste in urban centers through evidence-based federal and state regulation have a direct and immediate impact on health outcomes. Public/private partnerships could also facilitate access to affordable, healthier, nutrient-dense food and green spaces in under-resourced areas. Entrenched economic disadvantage must be addressed with high-quality public education, job skills, and taxation, and real estate and policing reforms, among others. The pandemic may have coalesced political will for paid sick leave and occupational retraining for entry-level, technology- and service-based jobs. Action should not be delayed as political will may be fleeting. States that refuse to expand Medicaid under the ACA are denying the most vulnerable working poor fair access to high-quality primary healthcare, a human right that is available in other high-income nations regardless of ability to pay, employment, or immigration status.

**Table 1:** Syndemic factors with proximal and distal solutions.

	**Policy solutions**	
**Syndemic factors**	**Proximal**	**Distal**
Disease concentration and interaction	• Increase number and dispersion of FQHC • Facilitate uptake of telemedicine to manage chronic health conditions • Reduce environmental hazards • Increase access to green spaces and affordable fresh food • Build national (i.e., state/federal cooperation) repository of infectious disease surveillance • Re-build public health infrastructure to employ primary healthcare providers and social workers (e.g., contact tracers, at-home care providers)	• Incentivize holistic, value-based, healthcare over per procedure approaches • Incentivize primary preventative care over high-technology specialty fields • Reduce carbon emissions through federal and state regulation • Federal mandate to report demographic (e.g., race/ethnicity) information for comparative analysis of disease burden in underserved communities
Economic disenfranchisement	• Paid sick leave • Increase affordable housing • Job retraining programs • Renew funding for public 2- and 4-year colleges	• Federal minimum wage • Strengthen Section 8 of the Housing Act for low-income and working families • Free college (academic and technical) and/or loan forgiveness programs for public secondary education
Barriers to healthcare	• Support community health centers for family and behavioral health • Expand Medicaid in all states • Reduce implicit bias in healthcare system	• Healthcare access for all • Increase diversity in healthcare provider pipeline
Racialized sociopolitical systems	• Increase implicit bias training and require college degree for police • Require police to reside in communities they serve • Require wrongful conduct lawsuits to be settled from pooled police pension funds, not taxpayer dollars	• Pass the John Lewis Voting Rights Act to buffer against a resurgence in voter suppression • Pass George Floyd Justice in Policing Act • Renew commitment to Civil Rights Act

Vulnerable patients from the lowest socioeconomic populations are often uninsured and seek healthcare through Federally Qualified Health Centers (FQHC) that provide crucial services for >25 million patients per year. Without FQHC, vulnerable populations would experience even worse disparities in access to healthcare, quality of care received, and health outcomes. The number of FQHC expanded under the ACA, but this expansion has not kept pace with growing racial, health, and economic disparities. Both the number and geographic dispersion of these centers should increase. Considering the disproportionate impact of COVID-19 on underserved communities, there should be a renewed commitment to FQHC with a substantial increase in funding to support more local community health centers, migrant health centers, healthcare for the homeless centers, public housing primary care centers, and centers that are dedicated to serving indigenous people. Furthermore, in a subsequent expansion of FQHC, policymakers should adopt a Health in All Policies (HiAP) approach which recognizes that health is created by a myriad of factors even beyond traditional healthcare and public health services to encompass social determinants of health, including safe and stable housing, food security, substance abuse treatment, and mental health services. A new generation of FQHC could be an important linchpin to reduce syndemic factors and the severity and course of infectious and chronic disease in marginalized communities.

The pandemic has revealed the harsh realities of systemic racism most visibly manifested by policing. We must rethink who is allowed to police and in what situations. For many duties, police should be replaced with mental health providers, social workers, and community-grounded professionals who are trained in culturally competent de-escalation skills. Police officers should reside in the communities they serve. When officers commit crimes, they must be prosecuted to the fullest extent of the law. Those who have been removed from one jurisdiction should not be allowed to seek employment in other localities. Independent boards representing members of the policed community must adjudicate complaints of non-criminal wrongdoing, not the police themselves.

## Discussion

To blunt the impact of COVID-19 on Black, Latino/Latina, and Native American communities, large-scale, systems-level, and multidisciplinary federal, state and local strategies are imperative. A lack of preparedness to manage the COVID-19 pandemic has shown vulnerabilities in healthcare and public health coordination and systems. National- and state-level responses to the pandemic amplify significant weaknesses in safety nets and public health infrastructure across various sectors of society. The utilization of syndemics theory, when supported by empirical evidence, can inform policymaking as the nation re-calibrates its healthcare and public health infrastructure priorities.

Too often, research, policy, and practice do not adequately incorporate the role that systemic racism plays in vulnerability to infectious and chronic diseases. Clinicians, academic scientists, and policymakers alike argue that racial disparities of this magnitude should be considered a civil rights issue that threatens life, longevity, and well-being. One federal official concisely opined “the burden of this disease has fallen on those who are least able to bear it.” The next generation of health policies will need to include syndemic perspectives to guide public health and healthcare. A coordinated federal-state response must include comprehensive clinically-informed, data-driven strategies that address historical injustices which have served as syndemic multipliers in BIPOC communities even as revitalized public health measures are deployed.

## Data Availability Statement

The original contributions presented in the study are included in the article/supplementary material, further inquiries can be directed to the corresponding author/s.

## Author Contributions

CW and SHV: conceptualization and writing. Both authors contributed to the article and approved the submitted version.

## Conflict of Interest

The authors declare that the research was conducted in the absence of any commercial or financial relationships that could be construed as a potential conflict of interest.

## Publisher's Note

All claims expressed in this article are solely those of the authors and do not necessarily represent those of their affiliated organizations, or those of the publisher, the editors and the reviewers. Any product that may be evaluated in this article, or claim that may be made by its manufacturer, is not guaranteed or endorsed by the publisher.
